# La fréquence des symptômes physiques dans les troubles anxio-dépressifs: étude transversale chez une population de 202 consultants psychiatriques

**DOI:** 10.11604/pamj.2018.31.149.15738

**Published:** 2018-10-29

**Authors:** Yassine Otheman, Asmaa Fakir, Mohamed Kadiri, Mohamed Zakariya Bichra

**Affiliations:** 1 Service de Psychiatrie, Hôpital Militaire d’Instruction Mohammed-V, Faculté de Médecine et de Pharmacie, Université Mohammed-V de Rabat, Maroc

**Keywords:** Frequency, physical symptoms, anxiety disorders, depressive disorders, Fréquence, symptômes physiques, troubles anxieux, troubles dépressifs

## Abstract

**Introduction:**

les symptômes physiques associés aux troubles anxio-dépressifs ont fait l'objet de plusieurs études depuis plusieurs décennies, vu leurs fréquences et leurs conséquences. Le but de notre étude est de préciser la fréquence des principaux symptômes physiques dans les troubles anxieux: trouble panique (TP), trouble anxiété généralisée (TAG) et troubles phobiques (TPh), ainsi que dans les troubles dépressifs: épisode dépressif majeur (EDM) dans le cadre d'un trouble dépressif.

**Méthodes:**

nous avons mené une étude transversale à visée descriptive, réalisée sur un échantillon de 202 consultants dans un service de psychiatrie.

**Résultats:**

l'âge moyen des patients est de 42 ans (19 à 70 ans), avec une légère prédominance féminine: 118 (58%). Les troubles anxio-dépressifs constatés sont: l'EDM: 113(56%), le TP: 61 (30.2%), le TAG: 55 (27.2%) et les TPh: 30 (14.9%). La fréquence des patients présentant de 2 à 5, et plus de 5 symptômes était respectivement de 15.9% et 39.6% dans les troubles dépressifs, et de 9.5% et 62.9% dans les troubles anxieux. Les symptômes les plus rapportés sont d'ordre cardiopulmonaire (75%), général (73.8%) et neurologique (65.8%).

**Conclusion:**

les symptômes physiques qui accompagnent les troubles anxio-dépressifs sont variables et souvent nombreux. Ils peuvent aggraver le pronostic de ces troubles psychiatriques en rendant difficile leur prise en charge. Un dépistage précoce de ces troubles, en portant une attention particulière à ces symptômes physiques, permettra de prévenir ces complications.

## Abstract (en)

**Introduction:**

physical symptoms associated with anxiety-depressive disorders have been the subject of several studies for several decades given their frequencies and their consequences. The aim of our study was to specify the frequency of the major physical symptoms of anxiety disorders such as panic disorder (PD), generalized anxiety disorder (GAD), phobic disorder (PhD) and depressive disorder (major depressive episode (MDD) in the framework of a depressive disorder).

**Methods:**

we conducted a cross-sectional descriptive study on a sample of 202 consultants in a department of psychiatry.

**Results:**

the average age of patients was 42 years (19-70 years), with a slight female predominance (118; 58%). Anxiety-depressive disorders included MDD (113;56%), PD (61;30.2%), GAD (55; 27.2%) and PhD (30;14.9%). Patients with 2 to 5 and more than 5 symptoms accounted for 15.9% and 39.6% respectively (depressive disorders), and 9.5% and 62.9% (anxiety disorders). The most reported symptoms were cardiopulmonary symptoms (75%), general symptoms (73.8%) and neurological symptoms (65.8%).

**Conclusion:**

physical symptoms associated with anxiety-depressive disorders are variable and often occur together. They can affect prognosis, making these psychiatric disorders difficult to treat. Early screening, paying particular attention to these physical symptoms, helps prevent these complications.

## Introduction

Les troubles anxio-dépressifs sont parmi les troubles mentaux les plus courants dans la population générale [1-3]. Ces troubles sont à l'origine d'une morbidité et d'un coût de santé élevé et constituent un fardeau pour les patients, leurs familles et la société [3-7]. Ces troubles peuvent se manifester de diverses façons. Les symptômes psychiques de type affectif, cognitif et comportemental sont les plus caractéristiques. Mais les symptômes physiques peuvent aussi faire partie du tableau clinique, et parfois même être au devant de la scène. Ces symptômes ne sont pas spécifiques, et restent extrêmement répandus dans le contexte de soins primaires de médecine générale et spécialisée en dehors de la psychiatrie [8, 9]. Ils peuvent survenir durant les différentes étapes d'évolution de ces troubles psychiatriques: inaugurer le tableau clinique, accompagner les symptômes psychiques dans la phase d'état ou compliquer leur évolution. Cela peut rendre parfois difficile le diagnostic, et retarder la prise en charge des patients qui consultent plusieurs spécialistes et subissent des examens et bilans répétés. Dans cette étude réalisée dans un service de psychiatrie d'un hôpital général de Rabat, nous allons déterminer la nature et la fréquence des principaux symptômes physiques, chez des patients consultant pour des troubles anxio-dépressifs, afin de mettre en évidence l'intérêt de penser à l'origine psychique de ces plaintes somatiques en médecine générale.

## Méthodes

Nous avons mené une étude transversale à visée descriptive qui a été réalisée au sein du Service de Psychiatrie de l'Hôpital Militaire d'Instruction Mohamed V de Rabat, qui est un hôpital général de catégorie universitaire. L'enquête s'est déroulée sur une période de 04 mois, et a concerné un échantillon de 202 patients qui ont consulté au service de psychiatrie, et chez qui les diagnostics de troubles anxieux ou dépressifs ont été posés selon les critères du DSM-5 (manuel diagnostique et statistique des troubles mentaux de l'association américaine de psychiatrie, dans sa 5^ème^ version) [10]. Les troubles anxio-dépressifs retenus sont: l'épisode dépressif majeur (dans le cadre d'un trouble dépressif), le trouble anxiété généralisée, le trouble panique et les troubles phobiques. Nous avons inclus dans cette étude les patients âgés de 18 ans et plus, ayant un diagnostic de trouble anxieux ou dépressif avec la présence des manifestations physiques. Alors que les patients ne présentant pas de symptômes physiques, ou dont les symptômes physiques sont dus à une affection médicale ou induits par des médicaments ou d'autres substances, ont été exclus de cette étude. Le questionnaire utilisé dans cette étude comprend deux parties: une première partie concerne les renseignements sociodémographiques et cliniques des patients: l'âge, le sexe, le niveau d'étude, l'activité professionnelle et le diagnostic du trouble dépressif ou anxieux; Et la deuxième comporte les renseignements concernant les symptômes physiques, notamment leur nature (l'organe ou l'appareil concerné) et leur fréquence. Nous avons choisi de les répertorier en deux groupes: de 2 à 5 symptômes et plus de 5 symptômes, et ce pour pouvoir comparer nos résultats avec ceux d'autres auteurs qui ont fait le même choix dans des études similaires. Les patients, précédemment informés sur les modalités de l'étude afin de pouvoir donner leur consentement oral, ont été interrogés pendant les jours de consultation, juste après leur séance avec leur psychiatre traitant. La durée de l'entretien était d'environ 10 minutes. L'aspect éthique a été pris en considération tout au long du déroulement de notre étude. Ainsi, plusieurs mesures ont été prises: présentation de l'étude et explication de ses objectifs et son intérêt, consentement des patients et respect de la confidentialité.

## Résultats

**Figure 1:**
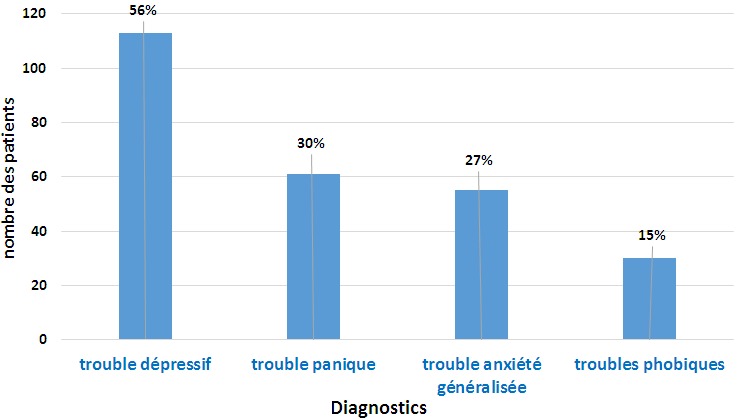
répartition des patients selon les diagnostics retenus

**Figure 2:**
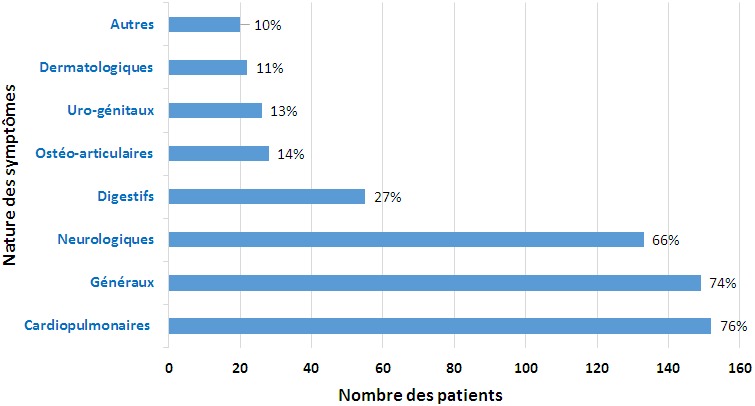
fréquence des symptômes physiques rapportés par les patients selon leur nature

L'âge moyen des patients est de 42 ans, avec des extrêmes allant de 19 à 70 ans. Nous avons noté la présence d'une légère prédominance féminine (Tableau 1). Sur le plan clinique, plus de la moitié des patients présentent un trouble dépressif, alors que la présence de plus d'un trouble a été retrouvée chez 56 patients (27,7%), le trouble anxieux le plus fréquent est le trouble panique (Figure 1). En ce qui concerne la fréquence des symptômes physiques dans notre étude, parmi les patients atteints de trouble dépressif, le pourcentage des patients présentant 2 à 5 symptômes est de 15.9%, et ceux présentant plus de 5 symptômes est de 39.6%, alors que parmi les patients atteints de troubles anxieux, il est respectivement de 9.5% et 62.9% (Tableau 2). Les symptômes physiques rapportés par les patients concernent plusieurs appareils, et sont de différentes natures (Tableau 3), les plus fréquents sont d'ordre cardiopulmonaire, général et neurologique (Figure 2), et leur nature est assez similaire dans les différents troubles psychiatriques, avec quelques différences pour les troubles phobiques où les troubles neurologiques sont moins fréquents (Tableau 4). Le dernier point étudié dans cette enquête est le moment de survenue des symptômes physiques par rapport aux symptômes psychiques. Ainsi, les symptômes physiques sont apparus en même temps que les symptômes psychiques chez 133 patients (66%), ils ont succédé au tableau psychique chez 47 patients (23%), alors qu'ils ont précédé l'apparition de ce tableau chez 22 patients (11%).

**Tableau 1:** caractéristiques sociodémographiques de la population étudiée

Caractéristiques	Nombre (pourcentage)
Age (ans)	42 ans [19-70]
18-40*	104 (51.5%)
41-60*	91 (45%)
>60*	7 (3.5%)
Sexe	
Féminin	118 (58%)
Masculin	84 (42%)
Profession	
Actifs	108 (53.5%)
Inactifs	94 (46.5%)
Niveau d’étude	
Illettrés	47 (23%)
Primaire	35(17%)
Secondaire	58 (29%)
Supérieur	62 (31%)

**Tableau 2:** nombre des symptômes physiques associés aux troubles anxio-dépressifs

Nombre de symptômes	Trouble dépressif	Trouble panique	Trouble anxiété généralisée	Troubles Phobiques
2 à 5	32 (15.9%)	8 (4%)	9 (4.5%)	2 (1%)
Plus de 5	80 (39.6%)	53 (26.2%)	46 (22.8%)	28 (13.9%)

**Tableau 3:** principaux symptômes physiques rapportés par les patients selon leur nature

**Cardio-pulmonaires**	Précordialgies, oppression thoracique, étouffement, tachycardie, palpitations, dyspnée, lipothymie.
**Généraux**	Asthénie, mains et pieds froides ou humides, sécheresse buccale, trouble du sommeil, anorexie, pâleur.
**Neurologiques**	Vertige, migraine, céphalée, tremblement et secousses musculaires, picotements, paresthésie, déficit fonctionnel des membres, clonie palpébral,
**Digestifs**	Nausées, vomissements, gène ou douleur abdominale, épigastralgies, diarrhée, constipation, dysphagie, dyspepsie
**Ostéo-articulaires**	Lombalgie, dorsalgie, cervicalgie, arthralgie, douleur musculaire, douleur osseuse
**Uro-génitaux**	Dysménorrhée, pollakiurie, aménorrhée, diminution de libido, frigidité, impuissance
**Dermatologiques**	Prurit, urticaire, chute des cheveux
**Autres**	Épistaxis, acouphènes, troubles visuels,

**Tableau 4:** fréquence des symptômes physiques en fonction du trouble psychiatrique

Troubles psychiatriques	Nature des Symptômes physiques	n(%) N=202
**Troubles dépressifs**	*Cardiopulmonaires	81 (74.3%)
	*Généraux	73 (67%)
	*Neurologiques	71 (65.1%)
	*Digestifs	26 (23.9%)
	*Dermatologiques	22 (20.2%)
	*Ostéo-articulaires	17 (15.6%)
	*Uro-génitaux	10 (9.2%)
	*Autres	7 (6.4%)
**-Trouble panique**	*Cardiopulmonaires	50 (82%)
	*Généraux	48 (78.7%)
	*Neurologiques	39 (63.9%)
	*Digestifs	25 (41%)
	*Dermatologiques	0
	*Ostéo-articulaires	7 (11.5%)
	*Uro-génitaux	14 (23%)
	*Autres	13 (21.3%)
**-Trouble anxiété généralisée**	*Cardiopulmonaires	41 (74.5%)
	*Généraux	48 (87.3%)
	*Neurologiques	45 (81.8%)
	* Digestifs	10 (18.2%)
	*Dermatologiques	7 (12.7%)
	*Ostéo-articulaires	8 (14.5%)
	*Uro-génitaux	3 (5.5%)
	*Autres	3 (5.5%)
**-Troubles phobiques**	*Cardiopulmonaires	27 (90%)
	*Généraux	27 (90%)
	*Neurologiques	14 (46.7%)
	*Digestifs	13 (43.3%)
	*Dermatologiques	0
	*Ostéo-articulaires	5 (16.7%)
	*Uro-génitaux	7 (23.3%)
	*Autres	6 (20%)

## Discussion

Notre étude permet d'avoir un aperçu sur les principaux symptômes physiques accompagnant les troubles anxieux et dépressifs chez les patients consultant dans un service de psychiatrie. Ces symptômes physiques surviennent souvent en nombre, dépassant ici les 5 symptômes, notamment chez les patients anxieux (62.9% contre 9.5% pour les patients ayant deux à cinq symptômes physiques). Les troubles anxio-dépressifs présentent plusieurs défis aux praticiens. Leur diagnostic et leur prise en charge rapides sont une condition première pour pouvoir les gérer et réduire les risques qu'ils peuvent engendrer. Parmi les difficultés qui peuvent avoir un impact négatif sur le cours évolutif de ces troubles, la présence de symptômes physiques qui, de par leur intensité et fréquence, peuvent faire ignorer le trouble psychiatrique et retarder sa prise en charge [11, 12], et donc aggraver le pronostic en général pour les patients [13-15]. En effet, les patients ayant des troubles anxio-dépressifs, avec symptomatologie somatique, consultent souvent au début un médecin généraliste ou d'autres médecins spécialistes, et expriment plus facilement leurs symptômes physiques que psychiques [16-19]. Cela implique une sensibilisation des médecins généralistes et spécialistes aux différents aspects des troubles anxio-dépressifs, pour permettre un dépistage plus précoce et donc une prise en charge adaptée rapide et efficace, en évitant les surenchères de bilans et de traitements symptomatiques. La population étudiée dans notre enquête est féminine à 58%, cela rejoint les données de la littérature, qui confirment que les symptômes somatiques sont plus fréquemment rapportés dans la population féminine atteinte de troubles affectifs [20, 21]. Nos résultats confirment aussi la prédominance des symptômes cardiopulmonaires, généraux et neurologiques, ainsi que la fréquence des troubles digestifs dans tous ces troubles. Cela a été évoqué par plusieurs études ayant évalué la fréquence de ces symptômes au cours des troubles anxio-dépressifs. En effet, les symptômes cardiorespiratoires sont des symptômes d'appel de la crise d'angoisse aiguë, et les relations entre l'anxiété et la dépression d'une part et les perturbations cardiaques et respiratoires de l'autre, sont largement étudiées [22-25]. Les symptômes neurologiques, et plus particulièrement les céphalées, sont aussi très présents parmi les plaintes des patients anxio-dépressifs, et peuvent constituer le motif principal de consultation des patients [26]. Sur le plan digestif, les plaintes gastro-intestinales sont aussi largement associées aux troubles anxio-dépressifs, notamment la constipation, l'épigastralgie et les coliques [27]. Les patients de notre étude, et notamment ceux présentant des troubles anxieux, présentent surtout des tableaux cliniques avec plus de cinq symptômes physiques. Ceci a été également décrit dans plusieurs études qui ont évalué la présence de ces symptômes dans les troubles anxio-dépressifs [28-30]. Certaines limites méthodologiques ont été soulevées lors de la réalisation de ce travail, notamment le fait qu'il s'agit d'une étude transversale portant sur le recueil d'informations lors d'un entretien unique se basant sur les données rapportées par le patient de façon subjective, avec la possibilité d'oubli de certains symptômes

## Conclusion

Les symptômes physiques accompagnant les troubles anxio-dépressifs sont de nature variable, notamment d'ordre cardio-pulmonaire, général et neurologique. Ils surviennent souvent en nombre, en particulier dans les troubles anxieux. Leur impact négatif sur le pronostic des patients par le biais du retard diagnostique qu'ils provoquent, implique une réflexion sur les moyens permettant un dépistage précoce. Cela permettra d'éviter les escalades d'investigations paracliniques et de traitements symptomatiques souvent inutiles voire nocifs.

### Etat des connaissances actuelles sur le sujet

- Les troubles anxio-dépressifs sont fréquents en pratique médicale générale;
- Le retard diagnostique aggrave le pronostic de ces troubles;
- Les symptômes physiques font partie du tableau clinique de ces troubles.

### Contribution de notre étude à la connaissance

- Les symptômes physiques des troubles anxio-dépressifs sont très variables et surviennent en nombre notamment pour les troubles anxieux;
- Les symptômes les plus fréquents sont d'ordre cardiopulmonaire, général et neurologique.

## Conflits d’intérêts

Les auteurs ne déclarent aucun conflit d’intérêts.
